# A Rare Case of Pancreatic Actinomyces

**DOI:** 10.14309/crj.0000000000000956

**Published:** 2023-01-20

**Authors:** Marguerite Poche, Kyle Liu, Codey Pham, Shilpa Jain, Robert Sealock

**Affiliations:** 1 School of Medicine, Baylor College of Medicine, Houston, TX; 2 Department of Medicine, Baylor College of Medicine, Houston, TX; 3 Department of Pathology and Immunology, Baylor College of Medicine, Houston, TX; 4 Section of Gastroenterology and Hepatology, Baylor College of Medicine, Houston, TX

**Keywords:** pancreas, Actinomyces, mass, GI

## Abstract

*Actinomyces israelii* is a filamentous, gram-positive anaerobic bacterium commonly found in the upper gastrointestinal tract, colon, and female genital tract. Rarely, actinomycosis can infect the pancreas most commonly after pancreatic instrumentation, often mimicking malignancy. We describe a case of a 26-year-old woman who presented with epigastric abdominal pain and nausea without prior pancreatic instrumentation. Abdominal imaging demonstrated a mass in the pancreatic head with fine-needle biopsy revealing *Actinomyces*. The patient was successfully treated with long-term antibiotic therapy resulting in resolution of the mass. Our case emphasizes the importance of considering nonmalignant etiologies in the differential diagnosis of pancreatic masses.

## INTRODUCTION

*Actinomyces israelii* is a filamentous, gram-positive anaerobic bacterium that is commonly found in the upper gastrointestinal tract, colon, and female genital tract.^1^ In rare situations, *Actinomyces* can infect the pancreas, especially after previous pancreatic stenting or pancreatic surgery.^2^ *Actinomyces* typically causes a granulomatous, fibrotic disease that presents with nonspecific symptoms, which can mimic malignancy leading to inappropriate and unnecessary treatment. We report a case of actinomycosis of the pancreatic head in a patient without a history of pancreatic instrumentation or surgery.

## CASE REPORT

A 26-year-old Hispanic woman with Down syndrome, type 2 diabetes mellitus, hypothyroidism, and obesity (body mass index 32 kg/m^2^) presented to clinic with epigastric abdominal pain and nausea. She had no associated vomiting, constipation, diarrhea, dysphagia, odynophagia, hematochezia, melena, or weight loss. She had no history of pancreatitis, surgery, or pancreatic instrumentation. Her vital signs were within normal limits. Her physical examination was notable for tenderness to palpation in the epigastric region. A complete blood count and complete metabolic panel were normal. She was prescribed famotidine initially without resolution of symptoms.

**Figure 1.**
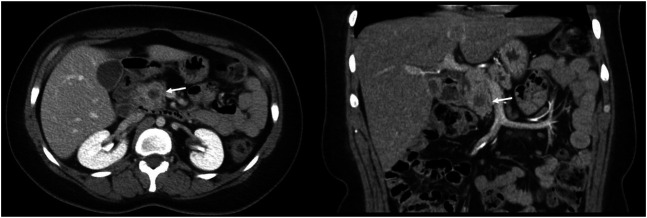
Computed tomography showing a solid 12 mm hypoenhancing mass in the inferior pancreatic head with associated pancreatic ductal dilation (5 mm) and mild hepatic steatosis.

**Figure 2.**
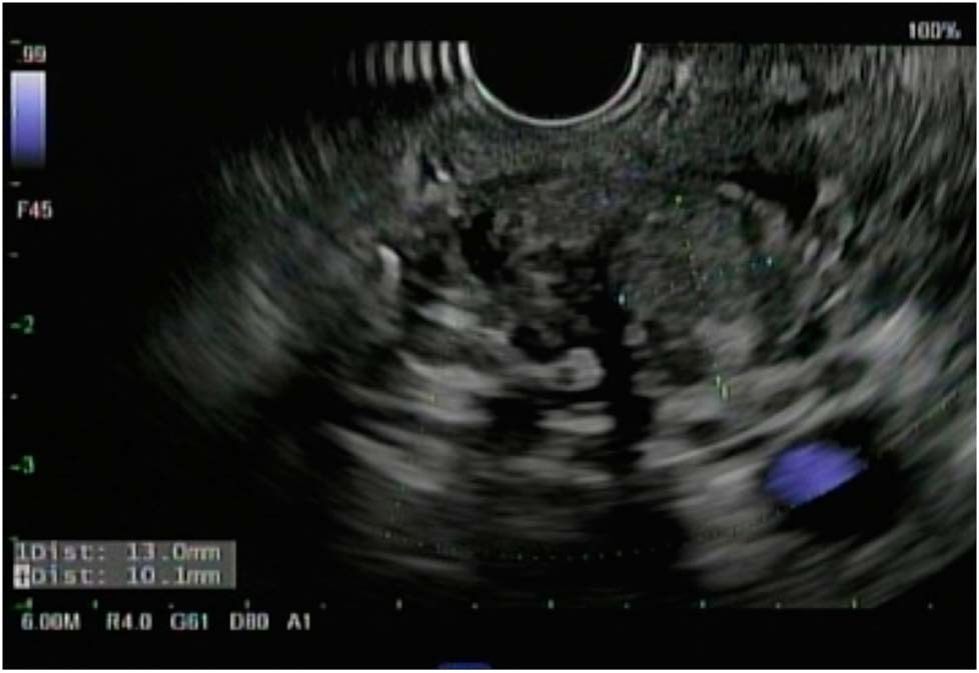
Endoscopic ultrasound showing a 15 mm mass in the pancreatic head.

**Figure 3.**
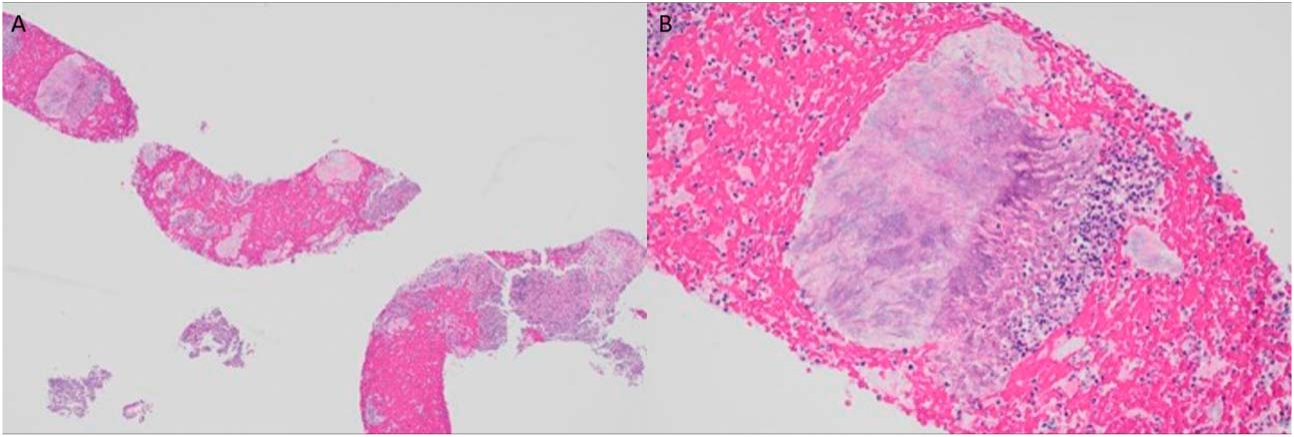
Hematoxylin and eosin stain of pancreatic biopsy at 40× showing marked acute inflammation with areas of necrosis and scant atrophic pancreatic acini (A) and at 200× showing lobulated structures surrounded by inflammatory cells, predominantly polymorphonuclear cells, morphologically consistent with actinomyces infection (B).

**Figure 4.**
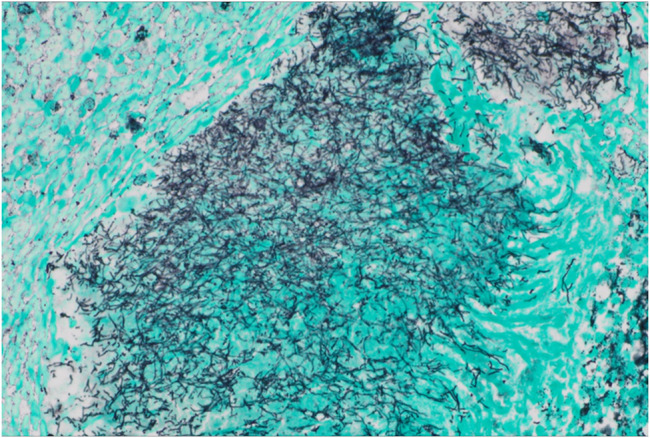
Grocott-Gomori methenamine silver stain at ×200 of pancreatic biopsy showing the filamentous bacterium while negative for acid-fast bacilli.

She returned 1 month later with persistent abdominal pain. An abdominal ultrasound revealed a dilated pancreatic duct. Abdominal computed tomography with intravenous contrast was subsequently performed demonstrating a solid 12 mm hypoenhancing mass in the inferior pancreatic head with associated pancreatic ductal dilation (5 mm) and mild hepatic steatosis (Figure 1). Endoscopic ultrasound (EUS) showed a 15 mm mass in the pancreatic head, and 4 ultrasound-guided fine-needle biopsies were obtained (Figure 2). Notably, the biopsies from the head of the pancreas demonstrated marked acute inflammation without dysplasia or malignancy. Hematoxylin and eosin stain showed lobulated structures surrounded by inflammatory cells, predominantly polymorphonuclear cells (Figure 3). Grocott-Gomori methenamine silver and Gram stains demonstrated a gram-positive, filamentous bacterium (Figure 4). Acid-fast bacillus stain was negative. The patient was diagnosed with *Actinomyces* infection of the pancreas and started on amoxicillin with an initial plan for 6–12 months of treatment.

**Figure 5.**
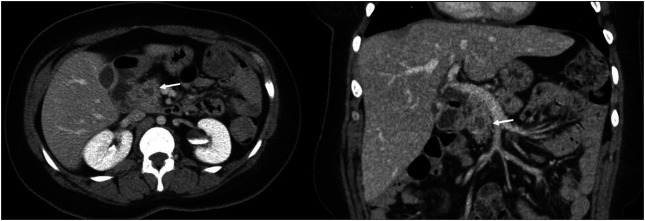
Computed tomography showing a stable 10 mm hypoenhancing mass of the inferior pancreatic head with persistent pancreatic ductal dilation (6 mm) after 9 months of amoxicillin therapy.

After 9 months of amoxicillin therapy, interval computed tomography demonstrated a stable 10 mm hypoenhancing mass of the inferior pancreatic head with persistent pancreatic ductal dilation (6 mm) (Figure 5). After 20 months of amoxicillin therapy, the patient had resolution of symptoms. Magnetic resonance imaging and subsequent EUS demonstrated resolution of the pancreatic head mass. The magnetic resonance imaging also revealed a 0.5 cm filling defect within the common bile duct. EUS and endoscopic retrograde cholangiopancreatography demonstrated choledocholithiasis of the distal common bile duct. Sphincterotomy with sphincteroplasty with stone extraction was performed with the placement of a prophylactic stent.

## DISCUSSION

Actinomycosis is a chronic, granulomatous infection that most commonly involves the orocervicofacial region.^1^ *Actinomyces* is a commensal organism often found in the oral cavity and gastrointestinal tract that rarely causes infection.^2^ Infection often requires breakdown of the mucosal barrier usually seen in the setting of trauma, surgery/instrumentation, or malignancy.^1^ The ileocecal region, liver, gallbladder, pancreas, and pelvis are all reported sites of abdominal actinomycosis, and infection can often mimic malignancy.^2,3^ Pancreatic actinomycosis, however, is an exceedingly rare site of infection. We were only able to identify 13 other documented cases of pancreatic actinomycosis in the literature.

There have been very few documented cases of pancreatic actinomycosis, and it is often preceded by pancreatitis, pancreatic surgery, or endoscopic stenting, although there are some cases without any preceding pancreatic manipulation.^1–3^ In this case, pancreatic actinomycosis developed in the absence of any prior pancreatic manipulation.

Visualization of gram-positive branching filaments by direct microscopy is the standard of diagnosis for actinomycosis.^4^ Grocott-Gomori methenamine silver staining can help distinguish *Actinomyces* but is not routinely performed on fine-needle biopsy specimens. It was performed reflexively in this case because of suspicion for actinomycosis based on the initial hematoxylin and eosin stain.

The standard treatment of actinomycosis involves high-dose long-term antibiotic therapy with penicillin or other beta-lactam antibiotics such as amoxicillin.^4,5^ Treatment duration is often 6–12 months if there is resolution of the lesion on serial imaging.^5^ In this case, there was persistence of infection on follow-up imaging and subsequent imaging was delayed, which led to a prolonged duration of antibiotic therapy. Surgical treatment is not standard but may be necessary when medical treatment is insufficient.^4^

This case of pancreatic actinomycosis in a patient with no known risk factors and nonspecific gastrointestinal symptoms demonstrates the importance of considering nonmalignant etiologies in the differential diagnosis of pancreatic masses. Autoimmune pancreatitis is another nonmalignant condition that may present as a pancreatic mass. EUS-guided fine-needle biopsy can provide a definitive diagnosis to guide management.

## DISCLOSURES

Author contributions: M. Poche and K. Liu wrote the article and reviewed the present literature. C. Pham revised the article for intellectual content and formatting. J. Shilpa provided pathologic images. R. Sealock revised the article and is the article guarantor.

Financial disclosure: None to report.

Informed consent was obtained for this case report.
